# Effect of Mahuang Fuzi and Shenzhuo Decoction on Idiopathic Membranous Nephropathy: A Multicenter, Nonrandomized, Single-Arm Clinical Trial

**DOI:** 10.3389/fphar.2021.724744

**Published:** 2021-10-18

**Authors:** Zhaocheng Dong, Haoran Dai, Yu Gao, Hanxue Jiang, Meiqi Liu, Fei Liu, Wenbin Liu, Zhendong Feng, Xiaoyan Zhang, Aijie Ren, Xiaolan Li, Hongliang Rui, Xuefei Tian, Guiming Li, Baoli Liu

**Affiliations:** ^1^ Beijing Hospital of Traditional Chinese Medicine Affiliated to Capital Medical University, Beijing, China; ^2^ Shunyi Branch, Beijing Traditional Chinese Medicine Hospital, Beijing, China; ^3^ Capital Medical University, Beijing, China; ^4^ Beijing University of Chinese Medicine, Beijing, China; ^5^ Beijing Chinese Medicine Hospital Pinggu Hospital, Beijing, China; ^6^ Yanqing Hospital of Beijing Chinese Medicine Hospital, Beijing, China; ^7^ Tangshan Fengrun Hospital of Traditional Chinese Medicine, Tangshan, China; ^8^ Zhangjiakou Hospital of Traditional Chinese Medicine, Zhangjiakou, China; ^9^ Department of Internal Medicine, Yale University School of Medicine, New Haven, CT, United States; ^10^ Department of Nephrology, Feicheng Mining Center Hospital, Tai’an, China

**Keywords:** idiopathic membranous nephropathy, Mahuang Fuzi and Shenzhuo Decoction, objective performance criteria, single-arm clinical trial, herbal medicine

## Abstract

**Objective:** To explore the clinical effect of Mahuang Fuzi and Shenzhuo Decoction on idiopathic membranous nephropathy.

**Methods:** This study is a multicenter, nonrandomized, single-arm clinical trial carried out as per the objective performance criteria, with the target being set at 35.0%. 184 cases of patients suffering from idiopathic membranous nephropathy with Shaoyin Taiyin syndrome were collected. These patients were treated with Mahuang Fuzi and Shenzhuo Decoction with a follow-up period of 3 years. The 24-hour urine protein and blood albumin were observed, and the remission rates of the patients were compared with the target.

**Results:** The mean follow-up time was 18 (12.5, 30) months, and the remission rate was 61.4%, which is a statistically significant difference from the target group of 35%. The remission rates for patients who had and had not used immunosuppressive therapy were 59.6 and 65.5%, respectively, but the difference was not statistically significant (*p* = 0.254). However, the albumin before the treatment and the course of treatment of the patients was significantly correlated with the disease remission (*p* < 0.05). However, the albumin before the treatment and the course of treatment of the patients was significantly correlated with the disease remission (*p* < 0.05). There were no significant changes in renal function before and after treatment, and no severe adverse events occurred during treatment.

**Conclusion:** Mahuang Fuzi and Shenzhuo Decoction have significant effects on idiopathic membranous nephropathy, and has the same effect on patients with membranous nephropathy who are newly treated as well as those who have been treated with immunosuppressive therapy without remission. In addition, the efficacy of this regimen is related to the albumin and the duration of the therapy, but not to 24-hour urine protein or other factors.

## Introduction

Idiopathic membranous nephropathy (IMN) is one of the pathological types of primary nephrotic syndrome (NS), with approximately 5–10 patients per million population (Ruggenenti et al., 2017). The prevalence of this disease in China is increasing year by year, second only to IgA nephropathy in primary NS (Xu et al., 2016). IMN is an immune-mediated disease caused by the deposition of IgG and complement components in the underlying epithelium of the glomerular capillary wall. About one-third of patients with this disease will develop the end-stage renal disease (ESRD) (Keri et al., 2019). In contrast, the remaining third of patients will experience clinical remission with immunosuppressive therapy (IST), while the remainder will experience spontaneous remission with continued stable renal function (Couser, 2017). Although IST is effective, it also has significant toxic side effects, such as those of cyclophosphamide, including hyperglycemia, myelosuppression, infection, infertility, and cancer (van den Brand, 2017). In addition, these drugs also have a recurrence rate after discontinuation (Fervenza et al., 2017). This presents opportunities and challenges for traditional Chinese medicine in the treatment of this disease. Mahuang Fuzi and Shenzhuo Decoction (MFSD) is a common prescription used by Baoli Liu to treat the Shaoyin Taiyin syndrome of IMN. This study is a multicenter, nonrandomized, single-arm, clinical trial, using the objective performance criteria method to explore the effect of MFSD on idiopathic membranous nephropathy.

## Methods

### Inclusion Criteria

1) Patients meeting the diagnostic criteria of IMN in modern medicine, diagnosed by pathology and light or electron microscopy (Couser, 2017).
2) Patients meeting the diagnostic criteria of Shaoyin Taiyin syndrome in traditional Chinese medicine (formulated based on the standard of *Interpreting Zhang Zhongjing Medicine* compiled by Feng Shilun). Primary symptoms include the following: aversion to cold, chills in the hands and feet, back pain, swelling, abdominal distension, loose or dry stools, soft tongue, thin greasy or slippery coated with water, and sunken pulse. Secondary symptoms include the following: fatigue and weakness, shortness of breath, laziness in words, sweating, scanty urination, and nighttime frequency. Patients with the above three primary symptoms and two secondary symptoms can be determined to have Shaoyin Taiyin syndrome (Feng and Zhang, 2011).
3) Patients who have complete case information and have been receiving traditional Chinese medicine treatment in our department for more than 8 months.
4) Patients aged between 16 and 80 years.
5) Patients with CKD1-3 (GFR>30 ml/min).

### Exclusion Criteria

1) Patients with CKD stage 3 or above.
2) Patients with other types of glomerular diseases.
3) Patients with confirmed secondary hepatitis B, systemic lupus erythematosus, tumors, and other factors.
4) Patients with acute central nervous system disease, severe gastrointestinal disease, history of HIV infection, history of mental illness, history of malignant tumor, and prohibition of immunosuppressive agents.
5) Patients with serious diseases, other organs dysfunctions, and life-threatening complications such as severe infection.
6) Pregnant or lactating women undergoing other clinical trials. Patients with any of the above conditions must be excluded from the study.

### Experimental Design and Sample Size Calculation

This study is a single-arm clinical trial with the objective performance criteria, and the efficacy of traditional Chinese medicine in the treatment of IMN was evaluated by setting a target value in advance. According to the literature, about one-third of patients with IMN are in spontaneous remission, clinical remission after IST, and no remission in ESRD (Couser, 2017). Because our team previously counted 108 outpatients with IMN, 50.0% of them did not respond after routine use of immunosuppressant (unpublished). In addition, during the progression of IMN, about one-third of the patients have spontaneous responses to effective immunosuppressive therapy and ineffective immunosuppressive therapy, meaning that, for newly treated IMN patients, the response rate to modern medical therapy is about 66.7%. Therefore, for the remaining 50.0% of the newly treated patients with IMN who came to our TCM outpatient department, only 66.7% of them could achieve remission if they continued to be treated with modern medical regimens. These remission patients accounted for 33.3% of our TCM clinics. Therefore, the target value of treatment was 35.0% (higher than the calculated value: 50% × 1/3 × 2 = 33.3%), with an expected target value of 45.0% to determine the efficacy. According to the requirement of single-arm clinical study with the objective performance criteria, the sample size was calculated and the shedding rate was designed to be 10%. The target value P_0_ = 0.35 and the target value P_1_ = 0.45 were set according to the formula used to calculate the sample size of the single-group target value test (US. FDA, 2013). The calculated results were 183, the designed shedding rate of this study was 10%, and the calculated test sample size was 202 cases in total.

### Case Shedding and Treatment

All subjects who complete the informed consent form and are screened for admission to the trial are referred to as shedding cases whenever or for any reason they withdraw, as long as the observation period specified in the protocol is not completed. The case records of exfoliation shall be kept, stating the cause of exfoliation, and converted to the final result based on the final test result.

### Treatment Options

The treatment group was treated on the basis of the KDIGO clinical practice guidelines issued in 2012 (Rojas-Rivera et al., 2019): 1) high-quality low protein and low phosphorus; 2) angiotensin-converting enzyme inhibitors (ACEIs) or angiotensin receptor blockers (ARBs) control blood pressure between 120-135/75-85 mmHg; 3) symptomatic diuretic, correct water, electrolyte, and acid–base balance; 4) anti-infection treatment for infected persons; and 5) conventional low molecular weight heparin anticoagulation for serum albumin below 25 g/L. The base prescription is MFSD, which contains Ma Huang (*Ephedra sinica* Stapf.) 15 g, Hei Fu Zi (*Aconitum carmichaeli* Debx.) 15 g (boiled first), Zhi Gan Cao (*Glycyrrhiza uralensis* Fisch.) 6 g, Gan Jiang (*Zingiber officinale* Rosc.) 20 g, Fu Ling (*Poria cocos* (Schw.) Wolf) 30 g, and Chao Bai Zhu (*Atractylodes macrocephala* Koldz.) 20 g. The main ingredients of these medicines in Chinese prescriptions are detailed in Supplementary Table S1. The dosage is as follows: 1 dose daily, boiled with 400 ml water, and taken in the morning and evening for 6–36 months. The distribution of herbs and collection of patient-related information will be conducted during the outpatient hours. Patients who are not seen in time will be followed up by telephone or online, and the initiative will be taken to mail the herbs to these patients if necessary.

### Observation Indicators and Methods

#### Main Curative Effect Index and Curative Effect Standard

The patient’s blood albumin and 24-hour urine protein quantification were monitored regularly to determine the remission (Kanigicherla et al., 2013). 1) Complete remission (CR): 24-h urine protein quantification <0.3 g, normal renal function and blood albumin, and disappearance of clinical symptoms. 2) Partial remission (PR): 24-h urine protein quantitative <3.5 h and a decrease of more than 50% from the base value, blood albumin increased before treatment, and normal renal function. 3) Non-remission (NR): the quantitative decrease of 24-h urine protein was less than 50% from the base value or deteriorated renal function, and the blood albumin was less than 30 g/L, without improvement in clinical symptoms.

#### Safety Standard

To observe whether hepatic and renal injury and other adverse reactions occurred in the treatment group during treatment.

### Statistical Analysis

The statistical analysis plan was developed according to the acquired data, and SPSS Statistics 20.0 software was adopted for analysis. The measurement data conforming to the normal distribution are expressed as the mean ± standard deviation, and the enumeration data and measurement data not conforming to the normal distribution are expressed as the median (interquaternary interval). In single-group analysis and comparison between two groups, the paired design quantitative data *t* test was used for measurement data conforming to the normal distribution, and the paired design sign rank-sum test or χ2 test was used for counting data and measurement data not conforming to normal distribution. Kaplan–Meier survival analysis was used to perform the log-rank test to compare the remission status of different groups, and survival curves were drawn. If the survival curves of two groups were intersected, the Breslow test would be used. Data relating to remission were selected to build the model, and remission or non-remission was taken as the dependent variable. The goodness-fit of the model was tested by the Hosmer–Lemeshow test. Based on this, a binary logistics regression analysis was conducted, and the ROC curve was plotted to calculate the cutoff value.

## Results

### Baseline Results

**FIGURE 1:**
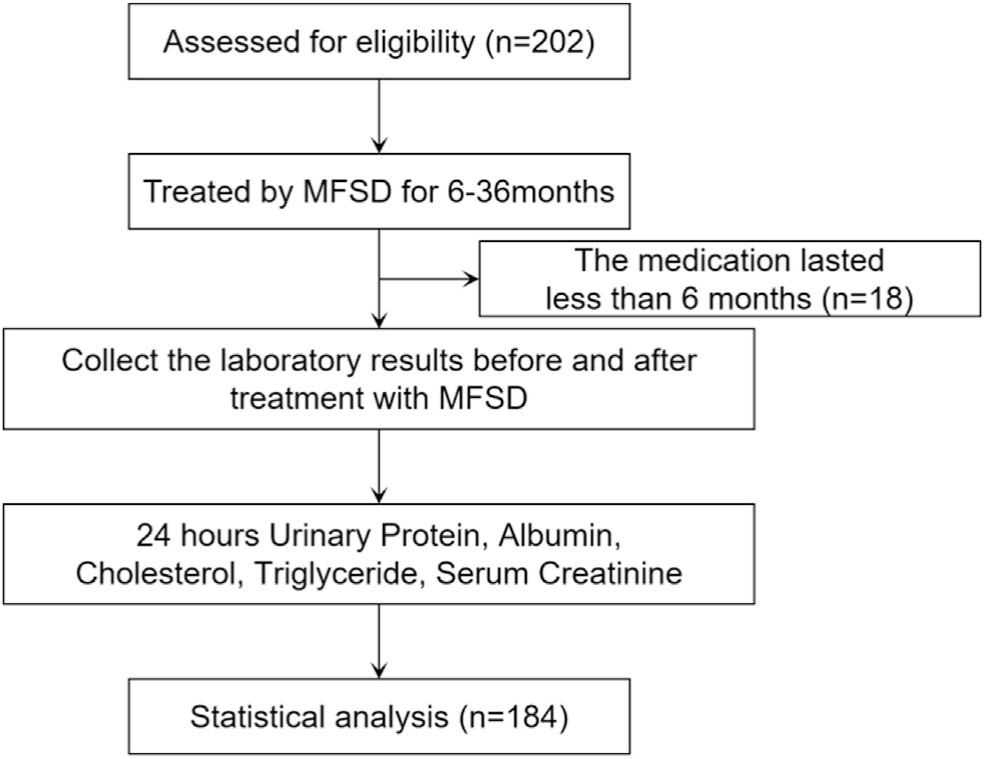
Study flow diagram.

A total of 202 IMN patients admitted to the Beijing Hospital of Traditional Chinese Medicine, Shunyi Branch of Beijing Hospital of Traditional Chinese Medicine, Yanqing Hospital of Beijing Hospital of Traditional Chinese Medicine, Tangshan Fengrun Hospital of Traditional Chinese Medicine, and Zhangjiakou Hospital of Traditional Chinese Medicine from September 2016 to January 2019 were selected as the research subjects. 18 patients who had been treated for less than 6 months were not included in the analysis based on clinical experience. Therefore, a total of 184 IMNs were included in the study (Figure 1), including 114 males and 70 females. The patients were aged 17–80 years, with a mean age of 50 (38, 60) years. The mean follow-up period was 18 (12.5, 30) months. 40 patients (21.7% of the total) were at low risk, 59 patients (32.1% of the total) were at moderate risk, and 85 patients (46.2% of the total) were at high risk. Among the patients studied, 126 patients had not been relieved by regular immunosuppressive therapy, and 58 patients had not been treated with immunosuppressive therapy. Of the patients who had previously received immunosuppressive therapy, 48 had received cyclophosphamide, 48 had received cyclosporine, 15 had received tacrolimus, 3 had received mycophenolate mofetil, 28 had received tripterygium glycosides, and 18 had received other regimens such as prednisone alone, leflunomide, and hydroxychloroquine. No patients had received the rituximab regimen. In addition, 106 patients had received only one treatment regimen, 11 patients had received two treatment regimens, and 9 patients had received three or more treatment regimens without remission (Supplementary Table S2).

Patients were grouped according to whether or not they had previously used immunosuppressants, and baseline levels were compared between the two groups. We identified 84 males and 42 females (mean 51 (40, 63) years old) with previous immunosuppressive therapy. Eighty-one patients, or 64.3% of the total, had nephrotic syndrome. 23.8% of patients were at low risk, 26.2% at medium risk, and 50.0% at high risk. The mean serum albumin was 25.61 ± 7.74 g/L, the mean 24-hour urine protein was 6.99 (4.00, 11.53) g/24 h, the mean cholesterol was 6.53 (5.25, 8.38) mmol/L, triglyceride was 2.20 (1.50, 3.23) mmol/L, serum creatinine was 72.00 (61.05, 93.75) μmmol/L, and the estimated glomerular filtration rate was 99.21 ± 35.84 ml/min. The mean follow-up period was 18 (13, 30) months. 44 patients had hypertension in the past, and 21 patients had diabetes. 30 males and 28 females (mean 47 (35, 59) years old) were not treated with immunosuppressive agents. 39 patients have nephrotic syndrome, accounting for 67.2% of the total. 17.2% of the patients were at low risk, 44.8% at medium risk, and 37.9% at high risk. The mean serum albumin was 25.38 ± 7.35 g/L, the mean 24-hour urine protein was 6.25 (4.53, 9.47) g/24 h, the mean cholesterol was 6.12 (5.55, 7.44) mmol/L, triglyceride was 2.20 (1.48, 3.51) mmol/L, serum creatinine was 64.40 (57.00, 77.60) μmmol/L, and the estimated glomerular filtration rate was 112.82 ± 25.14 ml/min. The mean follow-up period was 18.5 (12, 30) months. There were 25 patients with hypertension and 5 patients with diabetes. When comparing the two groups at baseline, we found that there was no statistical significance in the composition of gender between the two groups (*p* = 0.052). Patients who had received immunosuppressive therapy were older than those who had not, and the difference was statistically significant (*p* < 0.05). There were no significant differences in serum albumin, 24-hour urinary protein, total cholesterol, and triglyceride between the two groups (*p* > 0.05). The serum creatinine of patients who had received previous immunosuppressive therapy was significantly higher than that of patients who had not, and their glomerular filtration rate was significantly lower (*p* < 0.05). There was no significant difference in the risk ranking, treatment course, and medical history between the two groups (*p* > 0.05) (Table 1).

**TABLE 1:** Baseline characteristics of the subjects (grouped according to whether they had used immunosuppressive therapy in the past).

	Treated (*N* = 126)	Untreated (*N* = 58)	*p*-value
**Gender**			0.052
Male	84 (66.7%)	30 (51.7%)	
Female	42 (33.3%)	28 (48.3%)	
**Age**	51 (40, 63)	47 (35, 59)	0.041
**Nephrotic syndrome**	81 (64.3%)	39 (67.2%)	0.696
Albumin (g/L)	25.61 ± 7.74	25.38 ± 7.35	0.849
24-h urine protein (g/24 h)	6.99 (4.00, 11.53)	6.25 (4.53, 9.47)	0.507
Cholesterol (mmol/L)	6.53 (5.25, 8.38)	6.12 (5.55, 7.44)	0.570
Triglyceride (mmol/L)	2.20 (1.50, 3.23)	2.20 (1.48, 3.51)	0.602
**Risk ranking**			0.487
Low	30 (23.8%)	10 (17.2%)	
Medium	33 (26.2%)	26 (44.8%)	
High	63 (50.0%)	22 (37.9%)	
**Renal function**			
Serum creatinine (μmol/L)	72.00 (61.05, 93.75)	64.40 (57.00, 77.60)	0.008
eGFR (ml/min)	99.21 ± 35.84	112.82 ± 25.14	0.019
**Treatment course (month)**	18 (13, 30)	18.5 (12, 30)	0.773
**Medical history**			
Hypertension	44	25	0.287
Diabetes	21	5	0.145

### Efficacy Results

All the 184 included patients with IMN were followed up until December 2019, of which 113 met the criteria for remission and 71 did not, so the remission rate is 61.4% [95% C.I. 54.4–68.4%]. The target remission rate set by this regimen was 35.0%, the target remission rate was 45%, and the lower limit of the 95% confidence interval was greater than the target value and target value, proving that this regimen has significant efficacy (Supplementary Table S3). Of all patients included, the remission rate was 59.6%, the complete remission rate was 16.7%, the partial remission rate was 42.9% for those who had received previous immunosuppressive therapy, while for those who had not, these rates were 65.5, 10.4, and 55.1%, respectively. There was no significant difference in the remission rate between the two groups (*p* = 0.254).

For patients who had been treated for 6–12 months, these rates were 39.1, 6.5, and 32.6%, respectively. Among these patients, those who had received previous immunosuppressive therapy had a remission rate of 32.3%, the complete remission rate of 6.5%, and the partial remission rate of 25.8%, while these rates for those who had not received immunosuppressive therapy were 53.3, 6.7, and 46.7%, respectively. There was no significant difference in the remission rate between the two groups (*p* = 0.359).

For patients who took the drug for 13–24 months, the remission rate was 65.3%, complete remission rate 15.3%, and partial remission rate 50.0%. Among them, these rates for patients who had been regularly treated with immunosuppressive therapy were 68.7, 20.8, and 47.9%, respectively, while for those who had not, these rates were 58.3, 4.2, and 54.2%, respectively. There was no significant difference in the remission rate between the two groups (*p* = 0.122).

**FIGURE 2:**
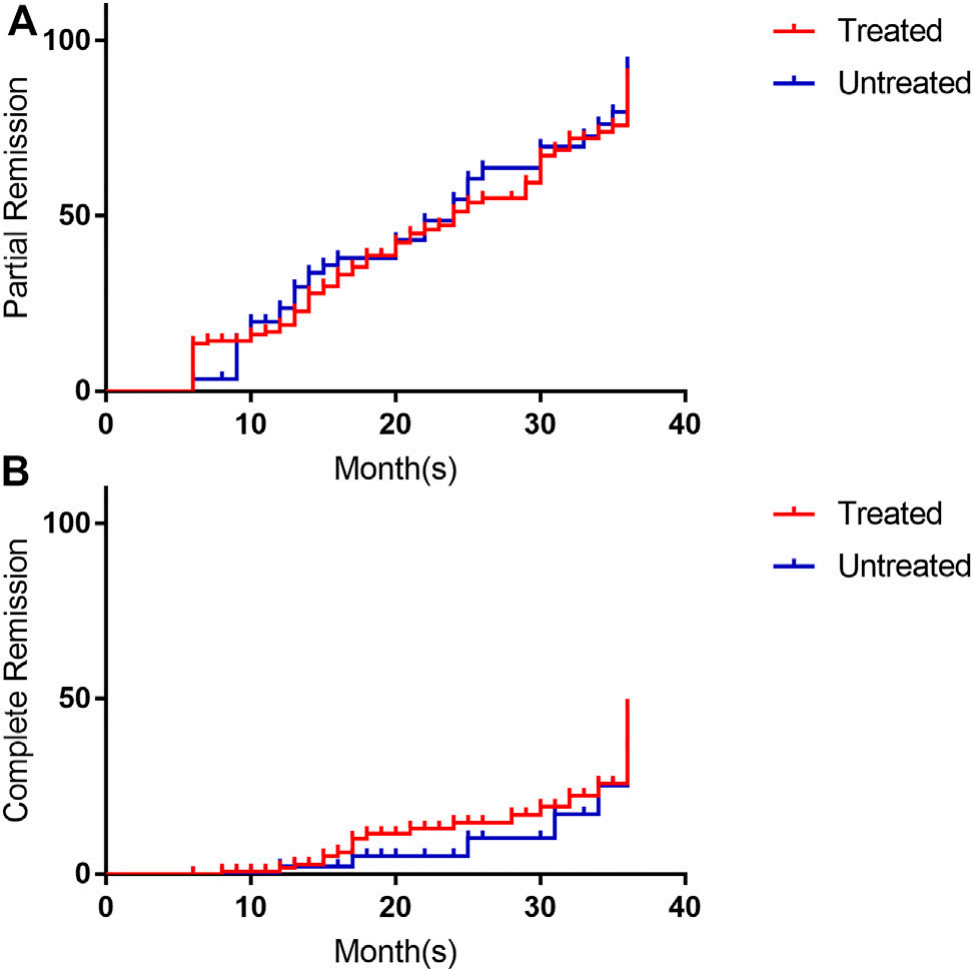
Survival analysis of remission rates (grouped by previous use of immunosuppressive therapy). **(A)** is the survival analysis of partial response rate, and **(B)** is the survival analysis of complete response rate. The red line is treated with immunosuppressive, the blue line is un-treated with immunosuppressive. Its annotation is consistent with the grouping words in Table 1.

For patients who took the drug for 25–36 months, these rates were 72.7, 19.7, and 53.0%. Among them, patients who had been regularly treated with immunosuppressive therapy had rates of 68.1, 19.1, and 48.9%, respectively, while those who had not been treated with immunosuppressive therapy had rates of 82.2, 21.1, and 63.2%. There was no significant difference in the remission rate between the two groups (*p* = 0.375; Table 2). The Kaplan–Meier survival analysis was performed based on the time to remission between the two groups, and the results showed that there was no statistically significant difference in the partial remission rate (*p* = 0.641) or complete remission rate (*p* = 0.346) between the two groups (Figure 2).

**TABLE 2:** Composition of remission rates (grouped by previous use of immunosuppressive therapy).

	All	Treated	Untreated	*p*-value
**All**				0.254
CR	27/184 (14.7%)	21/126 (16.7%)	6/58 (10.4%)	
PR	86/184 (46.7%)	54/126 (42.9%)	32/58 (55.1%)	
Both	113/184 (61.4%)	75/126 (59.6%)	38/58 (65.5%)	
**6–12 Months**				0.359
CR	3/46 (6.5%)	2/31 (6.5%)	1/15 (6.7%)	
PR	15/46 (32.6%)	8/31 (25.8%)	7/15 (46.7%)	
Both	18/46 (39.1%)	10/31 (32.3%)	8/15 (53.3%)	
**13–24 Months**				0.122
CR	11/72 (15.3%)	10/48 (20.8%)	1/24 (4.2%)	
PR	36/72 (50.0%)	23/48 (47.9%)	13/24 (54.2%)	
Both	47/72 (65.3%)	33/48 (68.7%)	14/24 (58.3%)	
**25–36 Months**				0.375
CR	13/66 (19.7%)	9/47 (19.1%)	4/19 (21.1%)	
PR	35/66 (53.0%)	23/47 (48.9%)	12/19 (63.2%)	
Both	48/66 (72.7%)	32/47 (68.1%)	16/19 (82.2%)	

After treatment with MFSD, the serum albumin increased to an average of 37.35 (30.75, 41.70) g/L, the 24-hour urinary protein quantified to an average of 2.23 (0.70, 4.10) g/24h, while the average values for the total cholesterol and the triglyceride decreased to 5.33 (4.49, 6.55) mmol/L and 1.69 (1.03, 2.45) mmol/L, respectively. These results were significantly different from those of the baseline before treatment, and were statistically significant (*p* < 0.001). In addition, the average serum creatinine was 69.20 (55.00, 83.03) μmol/L (*p* = 0.239), and the average estimated glomerular filtration rate was 107.36 ± 36.46 ml/min (*p* = 0.127). There was no significant difference between before and after treatment.

Patients were grouped according to whether they had used immunosuppressants in the past, and the results of various laboratory tests after treatment were compared between the two groups. For patients who had received immunosuppressive therapy, after treatment with MFSD, the average serum albumin increased to 37.85 (30.33, 41.73) g/L, the average 24-hour urinary protein level was 2.18 (0.71, 4.47) g/24 h, the average total cholesterol decreased to 5.38 (4.52, 6.75) mmol/L, and the average triglyceride was 1.69 (1.02, 2.66) mmol/L. These results were significantly different from those of the baseline before treatment, and were statistically significant (*p* < 0.001). After treatment, the serum creatinine decreased to an average of 70.90 (55.00, 87.80) μmol/L (*p* = 0.074), and the estimated glomerular filtration rate increased to an average of 104.73 ± 38.29 ml/min (*p* = 0.066). There was no statistical significance between the above combination and the difference before treatment.

For patients who had not received immunosuppressive therapy, after treatment with MFSD, the serum albumin increased to an average of 35.45 ± 7.81 g/L, the 24-hour urinary protein quantitative mean was 2.30 (0.56, 3.72) g/24 h, the total cholesterol decreased to an average of 5.04 (4.30, 6.43) mmol/L, and the triglyceride was 1.68 (1.08, 2.38) mmol/L. There were significant differences between the above results and the baseline before treatment, and were statistically significant (*p* < 0.05). The average serum creatinine after treatment was 67.90 ± 18.26 μmol/L (*p* = 0.385), and the average estimated glomerular filtration rate was 113.74 ± 31.08 (*p* = 0.901). There was no significant difference between the above combination and before treatment (Table 3).

**TABLE 3:** Test results before and after treatment (grouped according to the previous application of immunosuppressive therapy).

	**Prior treatment**	**Post-treatment**	*p* **-value**
**Albumin (g/L)**	24.95 (20.00, 31.00)	37.35 (30.75, 41.70)	<0.001
**24-hour urine protein (g/24** **h)**	6.57 (4.29, 10.21)	2.23 (0.70, 4.10)	<0.001
**Cholesterol (mmol/L)**	6.36 (5.39, 8.17)	5.33 (4.49, 6.55)	<0.001
**Triglyceride (mmol/L)**	2.20 (1.48, 3.30)	1.69 (1.03, 2.45)	<0.001
**Serum creatinine (μmol/L)**	71.00 (59.20, 85.10)	69.20 (55.00, 83.03)	0.239
**eGFR (ml/min)**	103.23 ± 33.54	107.36 ± 36.46	0.127
**Treated**	**Prior treatment**	**Post-treatment**	***p*** **-value**
**Albumin (g/L)**	25.00 (19.70, 31.85)	37.85 (30.33, 41.73)	<0.001
**24-h urine protein (g/24** **h)**	6.99 (4.00, 11.53)	2.18 (0.71, 4.47)	<0.001
**Cholesterol (mmol/L)**	6.53 (5.25, 8.38)	5.38 (4.52, 6.75)	<0.001
**Triglyceride (mmol/L)**	2.20 (1.50, 3.23)	1.69 (1.02, 2.66)	<0.001
**Serum creatinine (μmol/L)**	72.00 (61.05, 93.75)	70.90 (55.00, 87.80)	0.074
**eGFR (ml/min)**	99.21 ± 35.84	104.73 ± 38.29	0.066
**Untreated**	**Prior treatment**	**Post-treatment**	***p*** **-value**
**Albumin (g/L)**	25.38 ± 7.35	35.45 ± 7.81	<0.001
**24-h urine protein (g/24** **h)**	6.25 (4.53, 9.47)	2.30 (0.56, 3.72)	<0.001
**Cholesterol (mmol/L)**	6.12 (5.55, 7.44)	5.04 (4.30, 6.43)	0.002
**Triglyceride (mmol/L)**	2.20 (1.48, 3.51)	1.68 (1.08, 2.38)	0.001
**Serum creatinine (μmol/L)**	66.26 ± 14.34	67.90 ± 18.26	0.385
**eGFR (ml/min)**	112.82 ± 25.14	113.74 ± 31.08	0.901

### Prognostic Correlation Analysis

Patients were grouped according to whether they relieve or not. The following factors are compared between the two groups: gender, age, pathological type, nephrotic syndrome history, serum albumin before treatment, 24-h urinary protein before treatment, cholesterol before treatment, triglycerides before treatment, serum creatinine before treatment, the estimated glomerular filtration rate before treatment, immunosuppressive therapy history, and medical history. Factors with statistical differences were screened out. There were no significant differences in gender composition and mean age between the two groups (*p* > 0.1) when comparing the baseline levels of the two groups. The proportion of patients with nephrotic syndrome was significantly lower in the remission group than that in the non-remission group, and there was a significant difference between the risk ranking of the two groups (*p* < 0.001). Patients who had remission had significantly higher serum albumin levels and lower 24-hour urinary protein levels than those who did not (*p* < 0.001). The total cholesterol of patients with remission was significantly lower than that of patients without remission (*p* = 0.029). Patients who achieved remission had slightly lower triglycerides than those who did not, but the difference was not statistically significant (*p* = 0.183). In terms of renal function, there were no significant differences in serum creatinine and the estimated glomerular filtration rate between the two groups (*p* > 0.1). The mean treatment course for patients who achieved remission was significantly longer than that for those who did not (*p* = 0.001). Patients who had a previous history of hypertension achieved remission at a significantly lower rate than those who had not (*p* = 0.047). However, previous use of tacrolimus was associated with remission (*p* = 0.020) (Supplementary Table S4).

Previous histories of hypertension, tacrolimus, nephrotic syndrome, risk ranking, and treatment course were selected as Model A. The histories of hypertension, tacrolimus, serum albumin before treatment, 24-hour urinary protein before treatment, cholesterol before treatment, and course of treatment were used as Model B. Age, gender, history of hypertension, tacrolimus, serum albumin before treatment, 24-hour urinary protein before treatment, cholesterol before treatment, triglyceride before treatment, and the course of treatment were taken as Model C. According to the above, binary logistics regression is carried out. Based on Model A, we found that only previous use of tacrolimus, nephrotic syndrome, risk ranking, and treatment course were associated with remission from MFSD (*p* < 0.05). Using Model B, we found that only pretreatment serum albumin and duration of treatment were associated with remission from MFSD (*p* < 0.05), and tacrolimus medication history was no longer associated with remission (*p* = 0.224). Based on Model C, we found that serum albumin before treatment and the duration of treatment were still related with the remission of MFSD (*p* < 0.05; Table 4; Supplementary Table S5).

**TABLE 4:** Correlation between baseline predictors and mitigation outcomes in binary logistics regression analysis.

	Model A		Model B		Model C	
	Or (95%C.I.)	*p*-value	Or (95%C.I.)	*p*-value	Or (95%C.I.)	*p*-value
Gender					1.199	0.673
					(0.517–2.782)	
Age					1.000	0.975
					(0.971–1.031)	
Hypertension	0.609	0.163	0.801	0.583	0.783	0.565
	(0.303–1.224)		(0.363–1.768)		(0.340–1.801)	
Tacrolimus	0.269	0.040	0.435	0.224	0.409	0.209
	(0.077–0.944)		(0.114–1.666)		(0.102–1.647)	
Nephrotic Syndrome	0.346	0.034				
	(0.130–0.924)					
Risk Ranking	0.510	0.024				
	(0.284–0.915)					
Albumin (g/L)			1.096	0.008	1.093	0.012
			(1.024–1.174)		(1.020–1.171)	
24-h Urine Protein (g/24 h)			0.904	0.055	0.893	0.038
			(0.815–1.002)		(0.802–0.994)	
Cholesterol (mmol/L)			0.963	0.666	0.917	0.397
			(0.810–1.144)		(0.750–1.120)	
Triglyceride (mmol/L)					1.103	0.351
					(0.898–1.354)	
Treatment Course (Month)	1.074	<0.001	1.079	0.001	1.076	0.002
	(1.034–1.116)		(1.032–1.128)		(1.028–1.127)	

**FIGURE 3:**
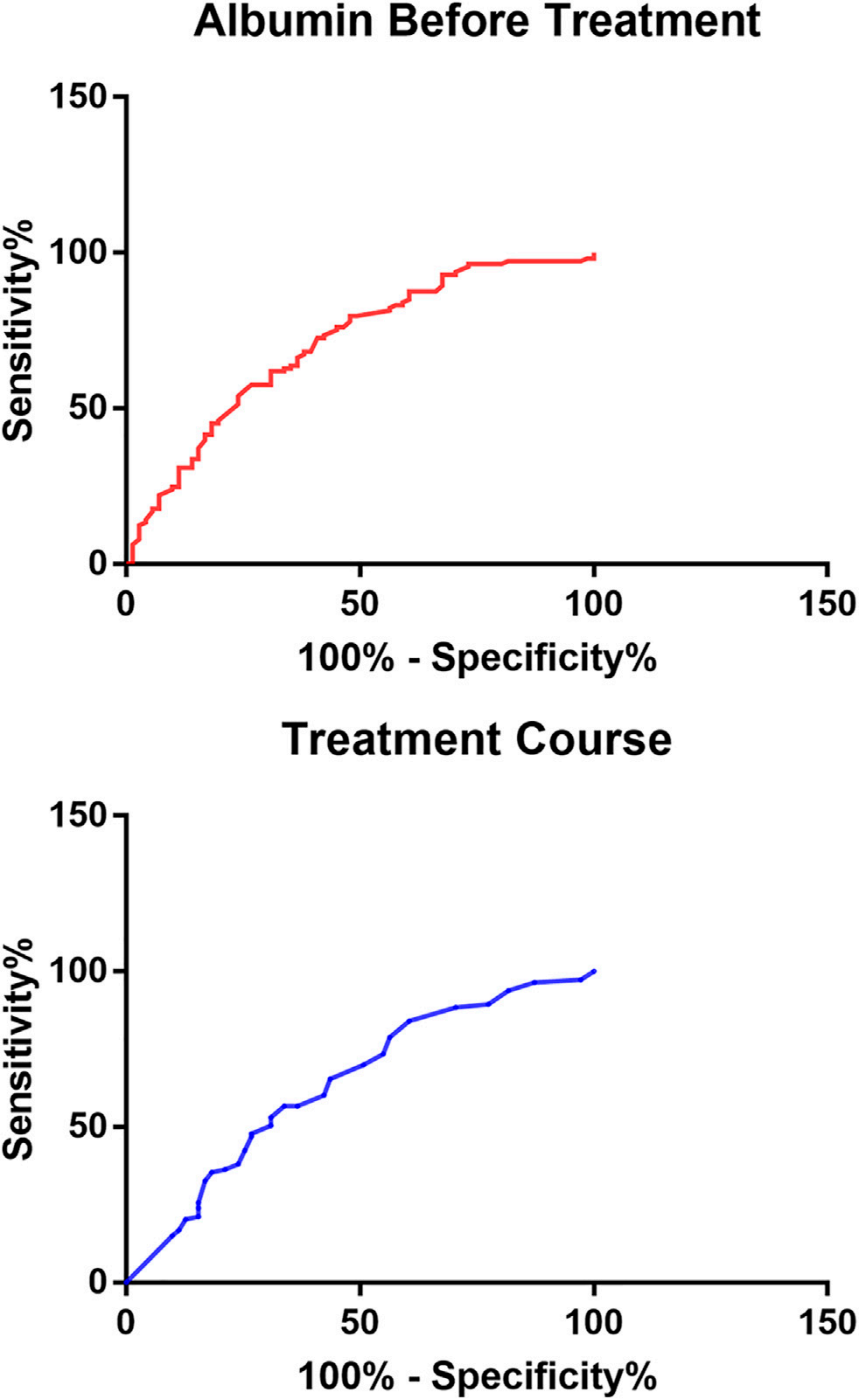
ROC curve of mitigation-related factors on mitigation prediction.

In order to further reflect the effects of serum albumin and 24-hour urinary protein quantification before treatment and treatment course on remission, we found through the ROC curve that the cutoff value of serum albumin before treatment was 22.95 g/L and the cutoff value of treatment course was 12.50 months, so 24-hour urinary protein quantification was difficult to be predicted whether patients were in remission or not (Figure 3).

### Safety Results

During the treatment period, no kidney damage (Table 3) and other adverse reactions, such as headache, nausea, and sweating, were observed in the treatment.

## Discussion

Although the treatment of IMN has been mature, the toxic side effects and recurrence rate are still important problems to be solved urgently (Liu et al., 2019). Moreover, for newly treated IMN patients, the most classic Italian protocol clinically used for the treatment of IMN had an overall remission rate of 58% at year 1, 54% at year 2, and 66% at year 3 (Ponticelli, 1992). The overall response rate of IMN after 1 year of cyclosporine treatment was 52% and that after 2 years of cyclosporine treatment was 20% (Fervenza, 2019). The overall response rates of IMN treated with rituximab were 60% at both year 1 and year 2 (Fervenza, 2019). The overall response rates of MFSD treatment were 53.3, 58.3, and 82.2% for year 1, year 2, and year 3, respectively. In short, the efficacy of herbal medicine for newly treated IMN patients is comparable to the current commonly used the immunosuppressive therapy regimen, and its total remission rate in the third year is much higher than that of the Italian regimen. In addition, there was no statistically significant difference between the efficacy of unremitted patients treated with immunosuppression and those who underwent initial MFSD treatment. These illustrate the advantages of the traditional Chinese medicine.

Although spontaneous remission has been reported in IMN patients with impaired renal function (Polanco et al., 2012), we classified them according to the risk level specified in the KDIGO guidelines and analyzed the number and use of previous immunosuppressive regimens. We found that 46.2% of 184 patients were at high risk, while 16.5% of these patients had unremission with two or more immunosuppressive regimens. However, we found no statistically significant correlation between previous use of immunosuppressants and whether remission was achieved with MFSD. These might prove that herbal medicine is effective in treating IMN, especially refractory IMN. On the other hand, 126 patients were treated with immunosuppressive therapy regularly, accounting for 68.5% of the total, which was higher than the 50% previously observed. This indicates that the target value of this study is accurately specified and the efficacy results have certain credibility.

In traditional Chinese medicine (TCM), there is no such disease named as “idiopathic membranous nephropathy,” as most patients have edema as the first symptom, so the disease can be classified as “edema disease” in TCM (Chen et al., 2013). Edema is due to fluid retention in the skin and subcutaneous tissue. This is a manifestation of the disturbance of the Qi-transformation function of the whole body (Wang et al., 2018). The location of this disease is on the surface. When we discuss its TCM pathogenesis, we must use modern medicine’s description of the clinical symptoms of IMN. IMN usually occurs in the elderly, with edema as the first symptom and thromboembolism (Alfaadhel and Cattran, 2015). “*Shang Han Lun*” records that “aversion to cold with fever belongs to the Yang syndrome, aversion to cold without fever belongs to the Yin syndrome.” The insidious onset of this disease, unlike other sudden onset of edema, coincides with the fact that IMN “belongs to Yin syndrome.” This is further supported by the fact that the disease is more commonly seen in the elderly (Kim et al., 2019). Thromboembolism indicates the presence of “blood stasis” in pathological products (Wang et al., 2017). Given it is Yin syndrome, the IMN patients have deficiency of Yang and excess of Yin, as the explanation in the “*Huang Di Nei Jing Su Wen*”: “the excess of Yin causes internal cold, warm qi is left while cold qi stays alone, thus causing blood coagulation.” To sum up, the pathogenesis of IMN is the deficiency of Yang and excess of Yin, and its pathological products are mainly water and blood stasis. According to Mr. Hu xishu’s syndrome differentiation system of *the six syndromes and the eight guiding principles*, or *Liu Jing Ba Gang* in Chinese, this disease belongs to the combination of exterior Yin syndrome and interior Yin syndrome, and is distinguished as Shaoyin Taiyin syndrome (Feng and Zhang, 2011). However, MFSD is used to treat the edema of Shaoyin Taiyin syndrome in “*Jin Gui Yao Lüe*,” namely, NS in modern medicine. Therefore, we compared the data in terms of whether or not there was NS.

The results of this study showed that MFSD could effectively treat IMN, and had no significant correlation with whether the presence of kidney syndrome, ineffectiveness of immunosuppressive treatment, gender, age, or previous medical history, *etc*. The following conclusions can be drawn from this study. First, this prescription can treat IMN that cannot be solved by current immunosuppressive regimens, indicating that the target of MFSD is different from or significantly exceeds that of traditional immunosuppressive regimens, which is a prototype for future ideas for new drug development. But the exact prescribing mechanism needs further study. Second, this course of treatment is the key to efficacy, which is stable after 12 months of administration. However, for each patient, the disease changes at each stage still need to be observed and analyzed to comprehensively evaluate whether there is any relapse of IMN treated by this prescription. Third, the efficacy of this prescription is related to the initial blood albumin, indicating the importance of albumin in evaluating the prognosis of patients. However, the disease remission still depends on whether the urine protein is reduced (Rojas-Rivera et al., 2019). We believe that with the development of IMN studies, the understanding of the blood albumin level will be deepened and be used as the evaluation criterion for efficacy. Finally, this subject is a single-arm clinical trial with objective performance criteria, not a randomized controlled trial, so there is a degree of bias in the data results. In the future, we will conduct a randomized controlled trial to comprehensively evaluate the difference in efficacy and safety between MFSD and IST, so as to demonstrate the advantages of TCM treatment more comprehensively.

## Data Availability

The raw data supporting the conclusion of this article will be made available by the authors, without undue reservation.

## References

[B1] Alfaadhel T. Cattran D. (2015). Management of Membranous Nephropathy in Western Countries. Kidney Dis. (Basel) 1, 126–137. 10.1159/000437287 27536673 PMC4934807

[B2] Chen Y. Deng Y. Ni Z. Chen N. Chen X. Shi W. (2013). Efficacy and Safety of Traditional Chinese Medicine (Shenqi Particle) for Patients with Idiopathic Membranous Nephropathy: a Multicenter Randomized Controlled Clinical Trial. Am. J. Kidney Dis. 62, 1068–1076. 10.1053/j.ajkd.2013.05.005 23810688

[B3] Couser W. G. (2017). Primary Membranous Nephropathy. Clin. J. Am. Soc. Nephrol. 12, 983–997. 10.2215/CJN.11761116 28550082 PMC5460716

[B4] Feng S. L. Zhang C. E. (2011). Interpretation of Zhang Zhongjing Medical Science - Prescription of Liu Jing. Beijing: People's Military Medical Press.

[B5] Fervenza F. C. Appel G. B. Barbour S. J. Rovin B. H. Lafayette R. A. Aslam N. (2017). Rituximab or Cyclosporine in the Treatment of Membranous Nephropathy. N. Engl. J. Med. 381 (1), 36–46. 10.1056/NEJMoa1814427 31269364

[B6] Kanigicherla D. Gummadova J. McKenzie E. A. Roberts S. A. Harris S. Nikam M. (2013). Anti-PLA2R Antibodies Measured by ELISA Predict Long-Term Outcome in a Prevalent Population of Patients with Idiopathic Membranous Nephropathy. Kidney Int. 83, 940–948. 10.1038/ki.2012.486 23364522

[B7] Keri K. C. Blumenthal S. Kulkarni V. Beck L. Chongkrairatanakul T. (2019). Primary Membranous Nephropathy: Comprehensive Review and Historical Perspective. Postgrad. Med. J. 95, 23–31. 10.1136/postgradmedj-2018-135729 30683678

[B8] Kim Y. Yoon H. E. Chung B. H. Choi B. S. Park C. W. Yang C. W. (2019). Clinical Outcomes and Effects of Treatment in Older Patients with Idiopathic Membranous Nephropathy. Korean J. Intern. Med. 34, 1091–1099. 10.3904/kjim.2018.139 31408925 PMC6718758

[B9] Liu D. Yang Y. Qing F. S. Hu B. Yu X. (2019). Risk of Infection with Different Immunosuppressive Drugs Combined with Glucocorticoids for the Treatment of Idiopathic Membranous Nephropathy: A Pairwise and Network Meta-Analysis. Int. Immunopharmacol. 70, 354–361. 10.1016/j.intimp.2019.03.002 30852290

[B10] Polanco N. Gutiérrez E. Rivera F. Castellanos I. Baltar J. Lorenzo D. (2012). Spontaneous Remission of Nephrotic Syndrome in Membranous Nephropathy with Chronic Renal Impairment. Nephrol. Dial. Transpl. 27, 231–234. 10.1093/ndt/gfr285 21624942

[B11] Ponticelli C. Zucchelli P. Passerini P. Cesana B. (1992). Methylprednisolone Plus Chlorambucil as Compared with Methylprednisolone Alone for the Treatment of Idiopathic Membranous Nephropathy. The Italian Idiopathic Membranous Nephropathy Treatment Study Group. N. Engl. J. Med. 327, 599–603. 10.1056/NEJM199208273270904 1640953

[B12] Rojas-Rivera Jorge. Enrique. Carriazo Sol. Ortiz Alberto. (2019). Treatment of Idiopathic Membranous Nephropathy in adults:KDIGO 2012, Cyclophosphamide and Cyclosporine A Are Out, Rituximab Is the New normal. Clin. Kidney J. 12, 629–638. 31583088 10.1093/ckj/sfz127 PMC6768298

[B13] Ruggenenti P. Fervenza F. C. Remuzzi G. (2017). Treatment of Membranous Nephropathy: Time for a Paradigm Shift. Nat. Rev. Nephrol. 13, 563–579. 10.1038/nrneph.2017.92 28669992

[B14] U. S. Food and Drug Administration (2009). Summary of Safety and Effectiveness Data (SSED). [2013-02-21] Availableat: http://www.fda.gov/downloads/AdvisoryCommittees/CommitteesMeetingMaterials/MedicalDevices/MedicalDevicesAdvisoryCommittee/CirculatorySystemDevicesPanel/UCM152225.pdf.

[B15] Van den Brand Jan. A. J. G. Ruggenenti P. Chianca A. Hofstra J. M. Perna A. Ruggiero B. (2017). Safety of Rituximab Compared with Steroids and Cyclophosphamide for Idiopathic Membranous Nephropathy. J. Am. Soc. Nephrol. 28 (9), 2729–2737. 10.1681/ASN.2016091022 28487395 PMC5576929

[B16] Wang X. Q. Wang L. Tu Y. C. Zhang Y. C. (2018). Traditional Chinese Medicine for Refractory Nephrotic Syndrome: Strategies and Promising Treatments. Evid. Based Complement. Alternat Med. 2018, 8746349. 10.1155/2018/8746349 29507594 PMC5817219

[B17] Wang Z. Tang Z. Zhu W. Ge L. Ge J. (2017). Efficacy and Safety of Traditional Chinese Medicine on Thromboembolic Events in Patients with Atrial Fibrillation: A Systematic Review and Meta-Analysis. Complement. Ther. Med. 32, 1–10. 10.1016/j.ctim.2017.03.006 28619293

[B18] Xu X. Wang G. Chen N. Lu T. Nie S. Xu G. (2016). Long-Term Exposure to Air Pollution and Increased Risk of Membranous Nephropathy in China. J. Am. Soc. Nephrol. 27, 3739–3746. 10.1681/ASN.2016010093 27365535 PMC5118492

