# Supplementary material for: Effect of Mahuang Fuzi and Shenzhuo Decoction on Idiopathic Membranous Nephropathy: A Multicenter, Nonrandomized, Single-Arm Clinical Trial
Source: Front Pharmacol. 2021 Oct 18;12:724744. doi: 10.3389/fphar.2021.724744 (PMC8558382; doi:10.3389/fphar.2021.724744)
Supplement: Supplementary file 1 [file DataSheet1.zip › Supplementary material 2.docx]

|  | **N=184** |
| --- | --- |
| **Gender** |  |
| Male | 114 (62.0%) |
| Female | 70 (38.0%) |
|  |  |
| **Age** | 50 (38, 60) |
|  |  |
| **Nephrotic Syndrome** | 120 (65.2%) |
| Albumin (g/L) | 25.53±7.60 |
| 24-hour Urine Protein (g/24h) | 6.57 (4.29, 10.21) |
| Cholesterol (mmol/L) | 6.36 (5.39, 8.17) |
| Triglyceride (mmol/L) | 2.20 (1.48, 3.30) |
|  |  |
| **Risk Ranking** |  |
| Low | 40 (21.7%) |
| Medium | 59 (32.1%) |
| High | 85 (46.2%) |
|  |  |
| **Renal Function** |  |
| Serum Creatinine (μmol/L) | 71.00 (59.20, 85.10) |
| eGFR (ml/min) | 103.23±33.54 |
|  |  |
| **Previous Treatment Regimens** |  |
| Glucocorticoid Alone | 14 |
| Cyclophosphamide | 48 |
| Cyclosporin A | 48 |
| Tacrolimus | 15 |
| Mycophenolate Mofetil | 3 |
| Tripterygium Glycosides | 28 |
| Others | 4 |
| Untreated | 58 |
|  |  |
| **Number of Previous Regimens** |  |
| 1 | 106 |
| 2 | 11 |
| 3 and more | 9 |
|  |  |
| **Treatment Course (Month)** | 18 (12.5, 30) |
|  |  |
| **Medical History** |  |
| Hypertension | 69 |
| Diabetes | 26 |
