# Supplementary material for: Effect of Mahuang Fuzi and Shenzhuo Decoction on Idiopathic Membranous Nephropathy: A Multicenter, Nonrandomized, Single-Arm Clinical Trial
Source: Front Pharmacol. 2021 Oct 18;12:724744. doi: 10.3389/fphar.2021.724744 (PMC8558382; doi:10.3389/fphar.2021.724744)
Supplement: Supplementary file 1 [file DataSheet1.zip › Supplementary material 3.docx]

| **Total** | **Remission** | **Non-remission** | **Remission Rate** | **95% C.I.** | |
| --- | --- | --- | --- | --- | --- |
|  |  |  |  | **Lower** | **Upper** |
| 184 | 113 | 71 | 61.40% | 54.40% | 68.40% |
