# Supplementary material for: Effect of Mahuang Fuzi and Shenzhuo Decoction on Idiopathic Membranous Nephropathy: A Multicenter, Nonrandomized, Single-Arm Clinical Trial
Source: Front Pharmacol. 2021 Oct 18;12:724744. doi: 10.3389/fphar.2021.724744 (PMC8558382; doi:10.3389/fphar.2021.724744)
Supplement: Supplementary file 1 [file DataSheet1.zip › Supplementary material 4.docx]

|  | **Remission (N=113)** | **Non-remission (N=71)** | **P-Value** |
| --- | --- | --- | --- |
| **Gender** |  |  | 0.348 |
| Male | 67 (59.3%) | 47 (66.2%) |  |
| Female | 46 (40.7%) | 24 (33.8%) |  |
|  |  |  |  |
| **Age** | 50 (37, 62) | 49 (38, 58) | 0.990 |
|  |  |  |  |
| **Nephrotic Syndrome** | 61 (54.0%) | 59 (83.1%) | ＜0.001 |
| Albumin (g/L) | 27.55±7.16 | 22.32±7.19 | ＜0.001 |
| 24-hour Urine Protein (g/24h) | 5.10 (3.17, 8.12) | 9.34 (6.24, 12.70) | ＜0.001 |
| Cholesterol (mmol/L) | 6.23 (5.27, 7.59) | 7.33 (5.40, 9.20) | 0.029 |
| Triglyceride (mmol/L) | 2.08 (1.46, 3.02) | 2.36 (1.55, 3.79) | 0.183 |
|  |  |  |  |
| **Risk Ranking** |  |  | ＜0.001 |
| Low | 32 | 8 |  |
| Medium | 45 | 14 |  |
| High | 36 | 49 |  |
|  |  |  |  |
| **Renal Function** |  |  |  |
| Serum Creatinine (μmol/L) | 70.00 (57.50, 83.20) | 71.00 (62.15, 91.00) | 0.211 |
| eGFR (ml/min) | 105.91±35.02 | 99.05±30.91 | 0.209 |
|  |  |  |  |
| **Treatment Course (Month)** | 22 (14, 31) | 16 (11, 25) | 0.001 |
|  |  |  |  |
| **Previous Treatment Regimens** |  |  |  |
| Glucocorticoid Alone | 7 | 7 | 0.363 |
| Cyclophosphamide | 29 | 19 | 0.869 |
| Cyclosporin A | 26 | 22 | 0.232 |
| Tacrolimus | 5 | 10 | 0.020 |
| Mycophenolate Mofetil | 0 | 3 | 0.028 |
| Tripterygium Glycosides | 18 | 10 | 0.735 |
| Others | 0 | 4 | 0.011 |
| Untreated | 38 | 20 | 0.439 |
|  |  |  |  |
| **Number of Previous Regimens** |  |  | 0.119 |
| 1 | 67 | 39 |  |
| 2 | 6 | 5 |  |
| 3 and more | 2 | 7 |  |
|  |  |  |  |
| **Medical History** |  |  |  |
| Hypertension | 36 | 33 | 0.047 |
| Diabetes | 14 | 12 | 0.394 |
