# Supplementary material for: Effect of Mahuang Fuzi and Shenzhuo Decoction on Idiopathic Membranous Nephropathy: A Multicenter, Nonrandomized, Single-Arm Clinical Trial
Source: Front Pharmacol. 2021 Oct 18;12:724744. doi: 10.3389/fphar.2021.724744 (PMC8558382; doi:10.3389/fphar.2021.724744)
Supplement: Supplementary file 1 [file DataSheet1.zip › Supplementary material 5.docx]

| **Variate** | **Assignment** |
| --- | --- |
| Remission | Non-remission=0; |
|  | Remission=1 |
|  |  |
| Age | Female=0; Male=1 |
|  |  |
| Hypertension | No=0; Yes=1 |
|  |  |
| Nephrotic Syndrome | No=0; Yes=1 |
|  |  |
| Risk Ranking | Low=1; Medium=2; High=3 |
