# Supplementary material for: Effect of Mahuang Fuzi and Shenzhuo Decoction on Idiopathic Membranous Nephropathy: A Multicenter, Nonrandomized, Single-Arm Clinical Trial
Source: Front Pharmacol. 2021 Oct 18;12:724744. doi: 10.3389/fphar.2021.724744 (PMC8558382; doi:10.3389/fphar.2021.724744)
Supplement: Supplementary file 1 [file DataSheet1.zip › Supplementary material 1.docx]

| Herb | Main Component | Target |
| --- | --- | --- |
| MA HUANG | Dihydro-Beta-Ionone | AGT, IL10, IL4, INS, LTA, PPP3R1, TGFB1, TNF |
|  | Piperitone | AGT, IL10, IL4, LTA, PPP3R1, TGFB1, TNF |
|  | Ephedrine | APOA1, GAA, IL10, IL4, IL6, INS, LTA, TNF |
|  | Lauric Acid | CCL2, IL10, PLA2G1B |
|  | D-Norpseudoephedrine | GAA, INS, TNF, YWHAZ |
|  | Norpseudoephedrine | GAA, TNF |
|  | Linolenic Acid | IL13, INS, PLA2R1, TNF |
|  | 1-Octanol | IL6 |
|  | Acetophenone | INS |
|  | Delta-Terpineol | LTA, TGFB1 |
| FU ZI | Deltaline | ALB, LTA |
|  | Para-Aminophenol | APOA1, IL10, IL4, IL6, LTA, TNF |
|  | Aconine | GAA |
|  | Deltamine | LTA |
| GAN CAO | Tetrahydropalmatine | AGT, IL6, INS, MC1R |
|  | Glycyrrhetinic Acid | CCL2, IL4 |
|  | Methylglyoxal | HPGDS |
|  | Dimethyl Sebacate | PLA2G1B |
|  | Umbelliferone | SOD2 |
| GAN JIANG | Alpha-Curcumene | AGT, GAA, IL10, INS, PLA2G1B |
|  | Zingiberone | AGT, IL10, IL4, INS, LTA, PPP3R1, TGFB1 |
| FU LING | Ergosterol | ALB |
|  | Ergotamine | IL13, INS |
|  | Hydrangeic Acid | IL4, IL6, PLA2G1B, TNF |
|  | Choline | PODXL1 |
| BAI ZHU | Hinesol | TGFB1 |
|  | Jurubine | TNF |
